# Supplementary material for: Effects of in vitro hemolysis and repeated freeze-thaw cycles in protein abundance quantification using the SomaScan and Olink assays
Source: bioRxiv. 2025 Apr 5:2024.09.21.613295. Preprint. [Version 3] doi: 10.1101/2024.09.21.613295 (PMC11956925; doi:10.1101/2024.09.21.613295)
Supplement: Supplement 4 [file media-4.pdf]

SUPPORTING INFORMATION

Effects of in vitro hemolysis and repeated freeze-thaw cycles in protein abundance quantification using the SomaScan and Olink assays

Julián Candia[*§], Giovanna Fantoni[§], Ruin Moaddel[§], Francheska Delgado-Peraza, Nader Shehadeh, Toshiko Tanaka, and Luigi Ferrucci

Intramural research Program, National Institute on Aging, National Institutes of Health, Baltimore, MD 21224, USA

§ Co-first authors
* Corresponding author: julian.candia@nih.gov

Table of Contents

Supplementary Figure 1: 96-well plate design showing the appearance and distribution of samples.
Supplementary Figure 2: Comparison of hemolysis effects between SomaScan and Olink probes mapped to shared RBC proteins.
Supplementary Figure 3: Comparison of SOMAmer rank metrics of hemolysis effects using different levels of data normalization.
Supplementary Table 1: Hemolysis effects of Olink probes mapped to hemoglobin and carbonic anhydrase proteins.
Supplementary Table 2: Hemolysis effects of SomaScan probes mapped to hemoglobin and carbonic anhydrase proteins.
Supplementary Data 1: Hemolysis Index (HI) measurements and mixed-effects model results to assess HI differences in hemolyzed vs non-hemolyzed samples.
Supplementary Data 2: List of 10,893 unique SOMAmer-UniProt ID pairs, along with protein annotations extracted from UniProtKB.
Supplementary Data 3: List of 1,476 unique Olink-UniProt ID pairs, along with protein annotations extracted from UniProtKB.
Supplementary Data 4: List of 1,893 unique SOMAmer-Olink-UniProt ID triplets, along with protein data extracted from UniProtKB.
Supplementary Data 5: Mixed-effects model results to assess the effect of hemolysis and freeze-thaw cycles on SomaScan's protein concentration measurements.
Supplementary Data 6: Mixed-effects model results to assess the effect of hemolysis and freeze-thaw cycles on Olink's protein concentration measurements.
Supplementary Data 7: Gene Set Enrichment Analysis for Olink and SomaScan probes affected by hemolysis and freeze-thaw cycles.
