# Supplementary material for: Effects of in vitro hemolysis and repeated freeze-thaw cycles in protein abundance quantification using the SomaScan and Olink assays
Source: bioRxiv. 2025 Apr 5:2024.09.21.613295. Preprint. [Version 3] doi: 10.1101/2024.09.21.613295 (PMC11956925; doi:10.1101/2024.09.21.613295)
Supplement: Supplement 9 [file media-9.pdf]

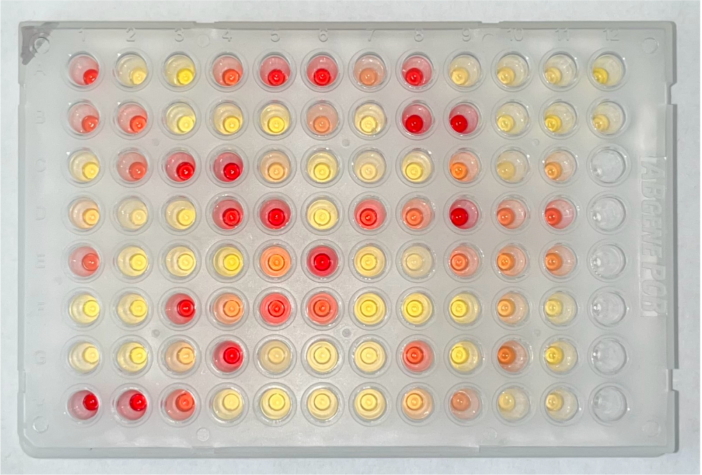

	1	2	3	4	5	6	7	8	9	10	11	12
**A**	s10_10_H	s12_20_nH	s04_3_nH	s08_3_H	s07_20_H	s14_10_H	s02_10_H	s07_10_H	s15_20_nH	s15_3_nH	s07_20_nH	s04_10_nH
**B**	s06_3_H	s15_3_H	s13_3_nH	s01_3_nH	s08_3_nH	s02_3_H	s10_20_nH	s09_20_H	s09_3_H	s13_10_nH	s14_3_nH	s07_3_nH
**C**	s01_20_nH	s06_10_H	s14_3_H	s09_10_H	s13_20_H	s11_20_nH	s06_3_nH	s08_20_nH	s04_10_H	s06_10_nH	s11_20_H	
**D**	s13_3_H	s02_20_nH	s12_3_nH	s07_3_H	s05_10_H	s09_3_nH	s10_20_H	s12_10_H	s03_20_H	s12_20_H	s15_10_H	
**E**	s06_20_H	s09_10_nH	s10_10_nH	s04_20_nH	s04_3_H	s03_10_H	s01_10_nH	s06_20_nH	s12_3_H	s01_3_H	s01_20_H	
**F**	s11_3_nH	s08_10_nH	s14_20_H	s01_10_H	s10_3_H	s08_20_H	s05_20_nH	s05_3_nH	s14_20_nH	s11_10_H	s13_20_nH	
**G**	s03_10_nH	s11_10_nH	s13_10_H	s05_20_H	s02_10_nH	s07_10_nH	s15_10_nH	s08_10_H	s05_10_nH	s04_20_H	s14_10_nH	
**H**	s03_3_H	s05_3_H	s15_20_H	s12_10_nH	s09_20_nH	s03_3_nH	s10_3_nH	s11_3_H	s02_20_H	s02_3_nH	s03_20_nH	

Supplementary Figure 1. 96-well plate design showing the appearance and distribution of samples. Samples are labeled by subject ID followed by the number of freeze-thaw cycles (3, 10, or 20) and a suffix indicating whether the sample was hemolyzed (H) or not (nH).
