# Supplementary material for: Effects of in vitro hemolysis and repeated freeze-thaw cycles in protein abundance quantification using the SomaScan and Olink assays
Source: bioRxiv. 2025 Apr 5:2024.09.21.613295. Preprint. [Version 3] doi: 10.1101/2024.09.21.613295 (PMC11956925; doi:10.1101/2024.09.21.613295)
Supplement: Supplement 10 [file media-10.pdf]

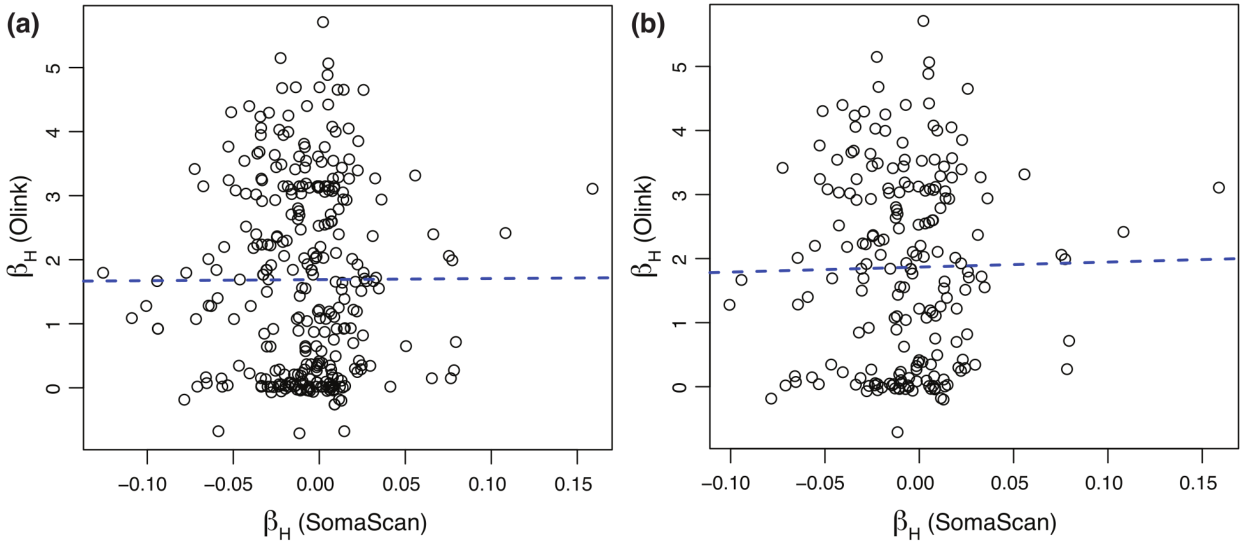

Supplementary Figure 2. Comparison of hemolysis effects between SomaScan and Olink probes mapped to shared RBC proteins. **(a)** Using 305 SomaScan / Olink probe pairs, the correlation is r = 0.004. **(b)** Using a subset of 199 SomaScan / Olink probe pairs (after excluding UniProt IDs that map to more than one SOMAmer or Olink probe), the correlation is r = 0.017. Linear fits are shown as blue dashed lines.
