# Supplementary material for: Effects of in vitro hemolysis and repeated freeze-thaw cycles in protein abundance quantification using the SomaScan and Olink assays
Source: bioRxiv. 2025 Apr 5:2024.09.21.613295. Preprint. [Version 3] doi: 10.1101/2024.09.21.613295 (PMC11956925; doi:10.1101/2024.09.21.613295)
Supplement: Supplement 11 [file media-11.pdf]

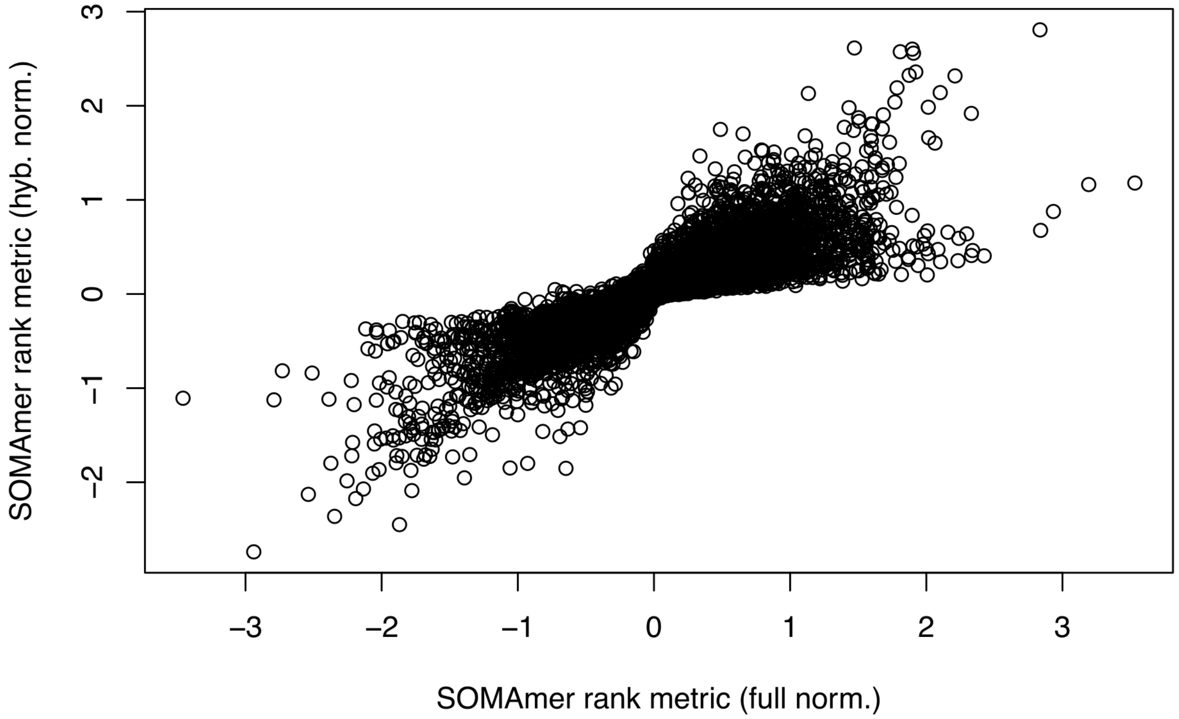

Supplementary Figure 3. Comparison of SOMAmer rank metrics of hemolysis effects using different levels of data normalization. SOMAmer rank metrics were defined as -log10(p-value_H)*sign(beta_H). The x-axis shows results obtained using the full normalization (*hybNorm.medNormInt.plateScale.calibrate.anmlQC.qcCheck.anmlSMP*). The y-axis shows results obtained using only the hybridization step (*hybNorm*). The correlation is r=0.867 (p-value<2.2e-16).
