# Supplementary material for: Effects of in vitro hemolysis and repeated freeze-thaw cycles in protein abundance quantification using the SomaScan and Olink assays
Source: bioRxiv. 2025 Apr 5:2024.09.21.613295. Preprint. [Version 3] doi: 10.1101/2024.09.21.613295 (PMC11956925; doi:10.1101/2024.09.21.613295)
Supplement: Supplement 12 [file media-12.pdf]

OlinkID	UniProt	Assay	Panel	(Intercept)	beta_H	p-value_H
OID21433	P09105	HBQ1	Oncology	0.66467556	3.75914889	3.40E-34
OID21078	Q9NZD4	AHSP	Neurology	1.43640565	1.21766222	8.33E-21

OlinkID	UniProt	Assay	Panel	(Intercept)	beta_H	p-value_H
OID20271	P07451	CA3	Cardiometabolic	1.360	3.416	9.76E-33
OID20409	P00915	CA1	Cardiometabolic	1.953	3.031	3.86E-54
OID21149	P00918	CA2	Neurology	2.695	1.193	1.40E-30

Supplementary Table 1. Hemolysis effects of Olink probes mapped to hemoglobin (top) and carbonic anhydrase (bottom) proteins.
