# Supplementary material for: Effects of in vitro hemolysis and repeated freeze-thaw cycles in protein abundance quantification using the SomaScan and Olink assays
Source: bioRxiv. 2025 Apr 5:2024.09.21.613295. Preprint. [Version 3] doi: 10.1101/2024.09.21.613295 (PMC11956925; doi:10.1101/2024.09.21.613295)
Supplement: Supplement 13 [file media-13.pdf]

SeqId	SeqIdVersion	SomaId	Target	UniProt	EntrezGeneID	EntrezGeneSymbol	Dilution	(Intercept)	beta_H	p-value_H
17137-160	3	SL005328	Beta-globin	P68871	3043	HBB	5.00E-01	3.716	-1.04E-01	0.495
18198-51	3	SL018342	HBAT	P09105	3049	HBQ1	2.00E+01	2.573	-8.40E-03	0.905
19774-8	3	SL009289	HBG2	P69892	3048	HBG2	5.00E-01	4.334	-1.16E-01	0.359
26273-24	4	SL017970	HBAZ	P02008	3050	HBZ	5.00E-01	3.804	-1.45E-02	0.899
32984-3	3	SL008874	HBG1	P69891	3047	HBG1	2.00E+01	3.920	-1.31E-01	0.444
4915-64	2	SL000836	Hemoglobin	P68871|P69905	3043|3039	HBB|HBA1	5.00E-01	3.762	-1.49E-01	0.412
6919-3	3	SL017970	HBAZ	P02008	3050	HBZ	5.00E-01	3.433	1.60E-02	0.872
6992-67	3	SL008001	HBD	P02042	3045	HBD	2.00E+01	2.860	1.66E-02	0.179
7965-25	3	SL018342	HBAT	P09105	3049	HBQ1	5.00E-01	3.425	3.17E-03	0.912
9025-5	3	SL010892	AHSP	Q9NZD4	51327	AHSP	2.00E+01	3.082	-6.05E-05	0.997

SeqId	SeqIdVersion	SomaId	Target	UniProt	EntrezGeneID	EntrezGeneSymbol	Dilution	(Intercept)	beta_H	p-value_H
3799-11	2	SL004867	Carbonic anhydrase III	P07451	761	CA3	5.00E-01	3.527	-7.24E-02	0.511
4969-2	1	SL004866	Carbonic anhydrase I	P00915	759	CA1	5.00E-01	4.645	-4.26E-02	0.502
4970-50	3	SL000339	carbonic anhydrase II	P00918	760	CA2	2.00E+01	2.679	-7.94E-04	0.940
4970-55	1	SL000339	carbonic anhydrase II	P00918	760	CA2	2.00E+01	2.385	2.18E-02	0.027

Supplementary Table 2. Hemolysis effects of SomaScan probes mapped to hemoglobin (top) and carbonic anhydrase (bottom) proteins.
